# Global effects of aging on the hemodynamic response function in the human brain

**DOI:** 10.21203/rs.3.rs-3299293/v1

**Published:** 2023-09-05

**Authors:** Nooshin J. Fesharaki, Amanda Taylor, Keisjon Mosby, Jung Hwan Kim, David Ress

**Affiliations:** Baylor College of Medicine; Baylor College of Medicine; Baylor College of Medicine; University of Texas Health Science Center; Baylor College of Medicine

## Abstract

In functional magnetic resonance imaging, the hemodynamic response function (HRF) is a transient, stereotypical response to local changes in cerebral hemodynamics and oxygen metabolism due to briefly (< 4 s) evoked neural activity. Accordingly, the HRF is often used as an impulse response with the assumption of linearity in data analysis. In cognitive aging studies, it has been very common to interpret differences in brain activation as age-related changes in neural activity. Contrary to this assumption, however, evidence has accrued that normal aging may also significantly affect the vasculature, thereby affecting cerebral hemodynamics and metabolism, confounding interpretation of fMRI aging studies. In this study, use was made of a multisensory stimulus to evoke the HRF in ~ 87% of cerebral cortex in cognitively intact adults with ages ranging from 22–75 years. The stimulus evokes both positive and negative HRFs, which were characterized using model-free parameters in native-space coordinates. Results showed significant age trends in HRF parameter distributions in terms of both amplitudes (e.g., peak amplitude and CNR) and temporal dynamics (e.g., full-width-at-half-maximum). This work sets the stage for using HRF methods as a biomarker for age-related pathology.

## Introduction

Functional magnetic resonance imaging (fMRI) is based on blood oxygenation level dependent (BOLD) contrast^1^. Briefly (< 4s) evoked neural activity gives rise to a transient, stereotypical BOLD response, known as hemodynamic response function (HRF). Assuming linearity, the HRF is used as an impulse response in fMRI data analysis. Models predict that the HRF corresponds to local changes in cerebral blood flow (CBF) and oxygen metabolism^2,3^. The usually dominant CBF component corresponds to neurovascular coupling (NVC). Accordingly, the HRF has potential to indicate neurovascular and neurometabolic integrity in the human brain^4,5^.

Neurovascular coupling is mediated by an elaborate microvascular architecture. A detailed structural characterization of this mesh in rodents showed a fairly regular hexagonal structure^6^. When neural activation occurs, a wave of vasodilation (*proximal integration*^7^) propagates from the capillary parenchyma to this pial mesh, creating a pressure fluctuation that drives the HRF. The cerebral microvasculature has a compliance that has been measured non-invasively in the human brain using diffuse correlation spectroscopy to be substantial, *C_p_* = 4.8 × 10^−4^ ml/mmHg/min/100g^8^. This compliance enables the pial mesh to store energy in the elasticity of pial arterioles. The pial mesh also stores kinetic energy in the form of moving blood. These features form the basis of our previous linear network modeling of the HRF^3,9,10^.

Evidence from animal models has accumulated showing that normal aging is associated with multifaceted vascular changes at molecular, cellular, and structural levels^11-13^. For instance, structural integrity of cerebral vessels can become compromised in aged rodent models^12,14,15^. Aging may also result in dysfunction of endothelial cells as well as proliferation and migration of vascular smooth muscle cells in arterioles^16^. Ultimately, increased vascular wall thickness but decreased vascular elasticity, affects conductance, causing vasomotility dysfunction^12,13^. More recently, impairment of pericyte dynamics has also been attributed into capillary remodeling in aged mice^17,18^. Additionally, aging is linked to vascular rarefaction and increased arterial tortuosity^19,20^. Advancing age may also increase large-artery stiffness, amplifying the dissipation of pulsatile energy in the heavily vascularized brain, causing microvasculature damage^21-23^.

In humans, age-related changes in vascular perfusion and metabolism of cerebral tissues have been quantified using imaging techniques such as arterial spin labeling MRI to measure CBF, and positron emission tomography to measure CMRO_2_^24-27^. There is a large body of evidence showing that aging correlates with reductions in both regional (e.g., temporal and parietal lobes) CBF and CMRO_2_, frequently with greater reductions in CBF than in CMRO_2_^24-26,28-31^. There is also evidence that both CBF and CMRO_2_ may remain intact in aged gray matter (GM) if the effect of brain atrophy is controlled^27^. Still other research has also shown the possibility for regional increases in both CBF and CMRO_2_ with age^32,33^. Thus, age-related changes in human resting CBF and CMRO_2_ remain controversial.

Using fMRI, many studies have linked BOLD signal variations in elderly subjects to age-related changes in neural activity^34-37^. In such age-related BOLD studies, it has been common to utilize a canonical HRF for BOLD-signal analysis, but this assumption is likely invalid with advancing age^38^. Clearly, there is a need to evaluate the how NVC and the HRF change with aging.

There have been several age-related studies of the HRF. Recently, West et al. (2019) regionally explored the characteristics of the BOLD HRF estimated using flexible basis sets to avoid assumptions on the shape of the HRF. They found significant differences in HRF parameters, particularly an increased time-to-peak and an decreased peak amplitude for older vs. younger subjects^38^. Their analysis, however, was restricted to a set of fairly coarse, atlas-defined brain regions. Additionally, some studies observe later time-to-peak with aging^39,40^ while others observe earlier time-to-peak with advancing age^41^. Yet another study reports no significant timing changes with age^42^. Other studies have concentrated on amplitude differences. Age-related decreases in amplitude in response to visual stimuli have been frequently reported^40,43-45^ and similarly for motor tasks^44,46,47^. Still others have observed no significant age differences in amplitude^34,41,42,48,49^. Thus, studies of age-related changes in the HRF have been inconsistent. Notably, all of these studies have suffered from two limitations. First, limited regional activation, so only a small subset of brain response was evaluated. Second, all used coarse spatial resolution that cannot resolve GM, confounding interpretation because of partial volume effects. HRF dynamics and amplitude vary between GM, white matter, and superficial vascular regions, and its reliability is highest in GM^50^.

Thus, it remains to fully characterize age-related changes in HRF dynamics and amplitudes across the whole of cerebral cortex GM. Such a study is important for three reasons. First, this knowledge is needed to interpret age-related neural changes to various tasks and stimuli. Second, such work can help us understand how age affects neurovascular and neurometabolic coupling. Most importantly, methods that broadly evoke the HRF across the brain have potential to provide a means to detect age-related changes in brain function and pathology.

Here, we evoked the HRF across the majority of cerebral cortex using the Speeded Audiovisual Sequence-following Task (SAST). The evoked HRFs have stereotypical amplitudes and stable dynamics in young healthy brains^51^, and are stable over time frames of up to three months^52^. These broadly evoked HRFs in healthy young brains provide a basis for comparison, a “fingerprint” against which age-related changes can be compared. As a first step in developing such methods, we used the SAST to evoke HRFs across cortex in 55 sessions that include subjects aged 22–75 years. We then investigated how the distributions of HRF amplitudes and dynamics across cortex change as a function of age. We find significant global effects in both amplitudes and dynamics that are consistent with known age-related microvascular changes.

## Results

**Figure 1:**
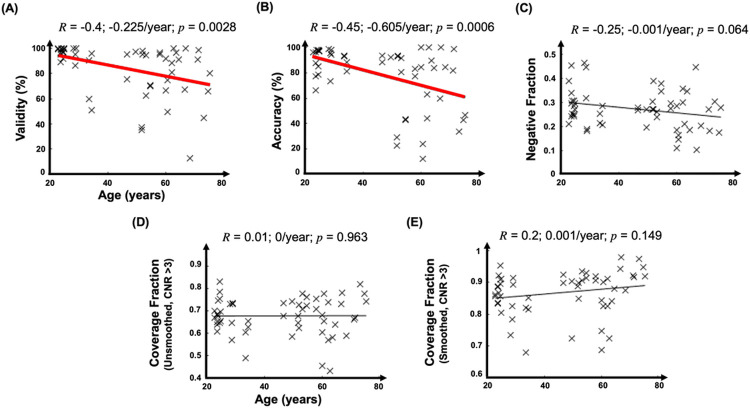
Correlations between age and (A) task-performance validity, (B) task-performance accuracy, (C) the fraction of cortex with negative HRFs, (D) the fraction of cortical coverage (CNR >3), and (E) the fraction of cortical coverage (CNR>3) with 8-mm FWHM smoothing (CNR>3). Pearson correlation coefficients (*R*) are shown for each feature. Significant (*p* < 0.05) correlations are marked by thicker regression lines.

Task performance was characterized by validity and accuracy. Valid tasks were those in which the subject pressed one of the assigned three buttons during the 2-s stimulus period. Average validity was 93 ± 10%. Accuracy, assessed for valid events, was the fraction of events where the subject pressed the correct button. Average accuracy was 79 ± 24%. Validity and accuracy decrease significantly with age (*R*=−0.40, and *R*=−0.45, respectively, Figs. 1A-B). Older subjects tended to respond too slowly, reducing both validity and accuracy.

At full resolution, the SAST effectively evoked strong HRFs (CNR > 3) across the majority of cerebral cortex. Coverage, the fraction of cortex with CNR > 3, range was 42–83%, mean of 68 ± 8%, where the variability noted is the SEM. Coverage was not affected by age (Fig. 1D). However, smoothing did increase cortical coverage substantially (Fig. 1E), with a coverage range of 69–97%, mean of 87 ± 7%.Consistent with our previous work^51^, a subset of activated cortex responded with nHRFs (negative fraction), with a range of 12–47%, mean 27 ± 8% across sessions. Additionally, there was a marginally significant trend for a decrease (*R*=−0.25, *p* = 0.064) in negative fraction with age (Fig. 1C). The negative fraction and its age trend were not significantly affected by smoothing.

**Figure 2:**
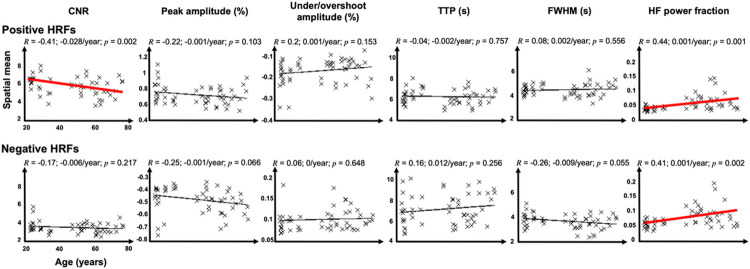
Correlations between age and the spatial mean of CNR, peak amplitude (PA), undershoot amplitude, time-to-peak (TTP), FWHM, and high-frequency power fraction (HFPF) for top, positive HRFs (pHRFs), and bottom, negative HRFs (nHRFs). Pearson correlation coefficients (*R*) are shown for each HRF parameter. Significant (*p*< 0.05) correlations are marked by thicker, red regression lines.

**Figure 3:**
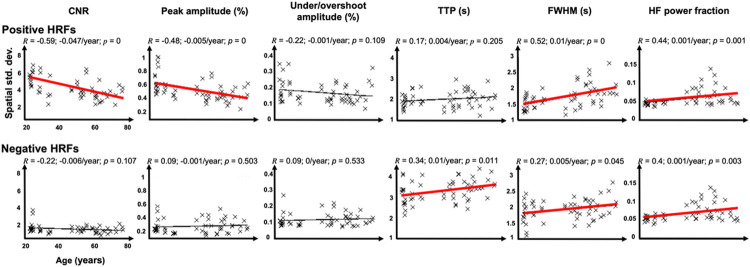
Correlations between age and the spatial standard deviation of CNR, peak amplitude (PA), undershoot amplitude, time-to-peak (TTP), FWHM, and high-frequency power fraction (HFPF) for top, positive HRFs (pHRFs), and bottom, negative HRFs (nHRFs). Pearson correlation coefficients (*R*) are shown for each HRF parameter. Significant (*p* < 0.05) correlations are shown by thicker, red regression lines.

**Figure 4:**
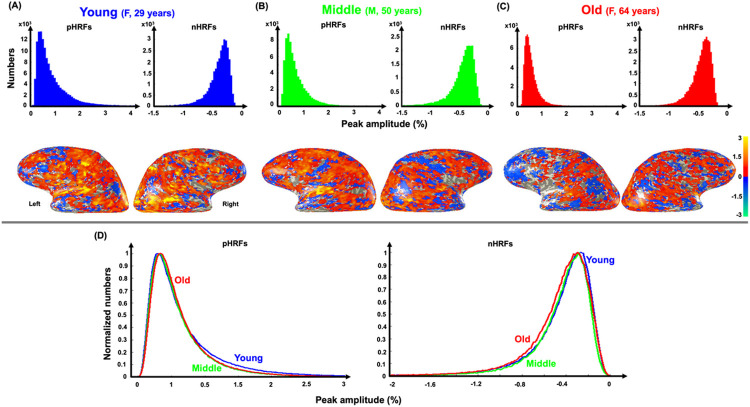
Example peak amplitude distributions of positive strong (CNR > 3) HRFs (pHRFs) and negative, strong (CNR >3) HRFs (nHRFs) of (**A**) 29-year-old female, (**B**) 50-year-old male, and (**C**) 64-year-old female. (**D**) Mean normalized distributions of PA for pHRFs (left) and nHRFs (right) are shown for young (23 subjects, blue), middle-age (14 subjects, green) and older (18 subjects, red) groups.

**Figure 5:**
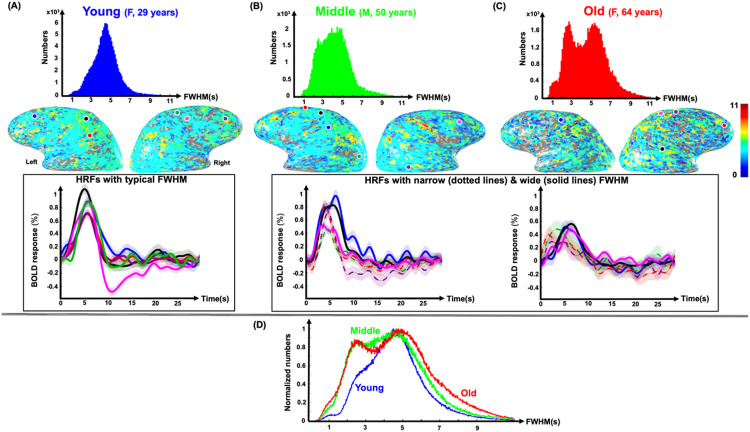
Example FWHM distributions of positive, strong HRFs (CNR > 3) of (**A**) 29-year-old female, (**B**) 50-year-old male, and (**C**) 64-year-old female. Corresponding HRF FWHM maps overlaid on gray-white interface surfaces are shown below each distribution. Average HRFs (5-mm gray-matter disk) in six example ROIs for each subject are shown in the next row. For middle-aged and older subjects, HRFs with example wide and narrow FWHM are shown by solid and dotted lines, respectively. Shaded regions show SEM. (**D**) Mean normalized distributions of FWHM for strong, positive HRFs are shown for young (23 sessions, blue), middle-age (14 sessions, green) and older (18 sessions, red) groups.

**Figure 6:**
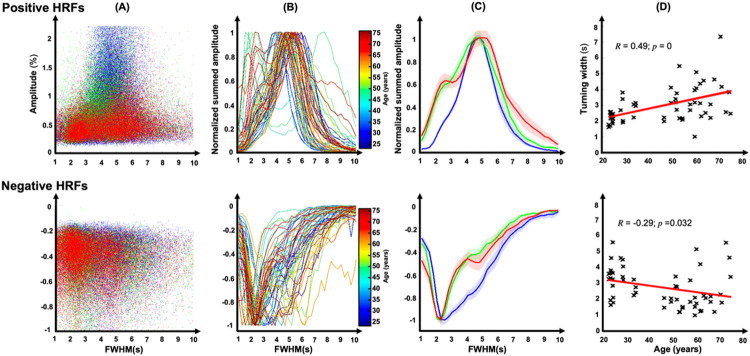
HRF tuning curves; upper row shows results for pHRFs, lower row for nHRFs. (A) Scatter plots of PA vs. FWHM for three example subjects (young, age 29, blue; middle, age 59, green; old, age 64, red). (B) Normalized tuning curves for all subjects, color coded by age. (C) Tuning curves averaged across the three age groups (young, blue; middle, green; old, red). Shaded regions are SEM across sessions. (D) Tuning widths vary significantly with age, increasing for pHRFs and decreasing for nHRFs.

**Figure 7:**
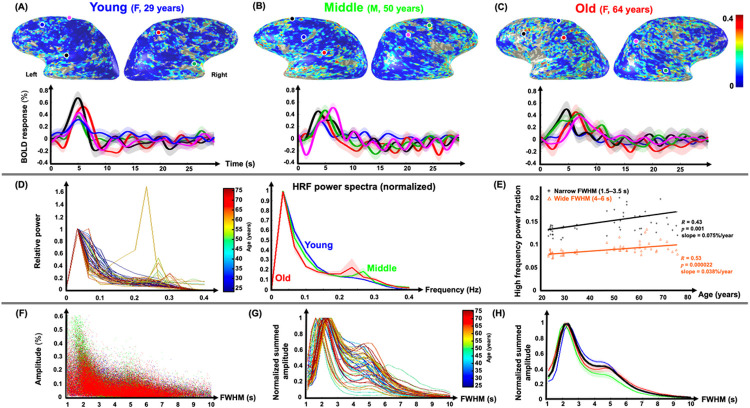
**Top:** HRF high-frequency power fraction (HFPF) maps overlaid on gray-white interface surfaces for (**A**) 29-year-old female (young), **(B)** 50-year-old male (middle), and **(C)**64-year-old female (old). **Second row:** Average HRFs (5-mm gray-matter disk) in five example ROIs with large HFPF for each subject. Shaded regions show SEM. **Third row:** (**D**) Left plot shows normalized HRF power spectra for all 55 sessions color coded by age. Right plot shows the average of normalized HRF power spectra across sessions in three age groups: young (blue), middle-aged (green), and older (red). (**E**) Correlations between HFPF and age for narrow (1.5–3.5 s) and wide (4–6 s) FWHM for all subjects are both strong and highly significant. **Bottom**: Tuning curves for HFPF. (**F**) Plotting of HFPF vs. FWHM shows a strong association between large HFPF and narrow FWHM. (**G**) This tuning peaks at a FWHM value of 2.3 s, with a lower peak or shoulder near a FWHM of 5 s. (**H**) This tuning pattern does not vary much with age. Shaded regions in all plots show SEM across sessions.

We first show the results of correlations between age and the spatial mean (Fig. 2) and the spatial variability (Fig. 3) of six HRF parameters: CNR, peak amplitude (PA), under/overshoot amplitude, TTP, FWHM, and HFPF. Next, we elaborate on those HRF parameters that significantly correlated with age (Figs. 4-7).

Figures 2 and 3 show correlations between age and the spatial means and standard deviations, respectively, of pHRF (first rows) and nHRF (second rows) parameters, respectively. In all plots, a thick red regression line indicates a significant (*p* < 0.05) age-related trend.

For pHRFs, mean CNR significantly (*p* = 0.002, *R* = −0.41), decreases with age. There is also a strong, significantly negative correlation between CNR spatial variability and age (*R* = −0.59, *p* ~ 0). For PA, there is a marginally significant and modest (*R*=−0.22, *p* = 0.10) drop in amplitude with age, which is consistent with the CNR drop. PA variability also significantly drops with age (*R* = −0.48, *p* = 0). Both the spatial mean and variability of undershoot amplitude did not significantly correlate with age, although there is trend for age-related increase in mean undershoot amplitude (*R* = 0.20, 0.001%/year, *p* = 0.15). The undershoot somewhat weakens with age (becomes less negative), which is consistent with the correlation between peak and undershoot amplitude previously reported.^51^

We tested for correlations between task performance and PA and CNR but found no significant trends. Task accuracy, for example, had age correlations of *R*=−0.083 (*p* = 0.55) for amplitude and *R* = 0.006 (*p* = 0.95) for CNR. Similarly weak and insignificant correlations were observed for the validity with mean PA and CNR, and likewise for the standard deviations of the PA and CNR distributions.

Dynamical parameters show three strong age-dependent correlations. First, the spatial variability of FWHM significantly increased with age (*R* = 0.52, p ~ 0). Additionally, there were substantial increases in both the mean and spatial variability of HFPF with age (*R* = 0.44, *p* = 0.001).

Spatial smoothing does affect these correlations (Figs. S1 & S2). Smoothing weakens the downward trend of CNR with age to −0.03/year (*p* = 0.063). The downward trend of mean PA and undershoot also both weaken toward insignificance. However, the significant dynamical trends in FWHM variability, and HFPF mean and variability were largely unaffected by smoothing.

For nHRFs, as shown in the second (bottom) rows of Figs. 3 and 4, our results did not show any significant age trends in the moments of the CNR or overshoot amplitude distributions. There was a marginal trend (*p* = 0.066) for a strengthening of mean PA (peak negative HRF amplitude becomes more negative with age). Dynamically, there were several significant trends. Similar to the pHRFs, mean HFPF increased significantly with age (*p* = 0.002). Likewise, nHRF spatial variability increased for FWHM (*p* = 0.045) and HFPF (*p* = 0.003). In addition, there was a significant trend for the spatial variability of TTP to increase with age (*R* = 0.34, 0.01/year, *p* = 0.011). The mean FWHM also had a marginally significant trend to decrease with age (*R* = 0.26, *p* = 0.055). Smoothing again had little effect on these trends for nHRFs (Figs. S1 & S2).

We further investigated the spatial variability of HRF PAs across three age groups of young, middle-age, and old. Figure 4 shows PA histograms of strong pHRFs and nHRFs for three example subjects, each representative of an age group: a 29-year-old female adult for the young group (Fig. 4A), a 50-year-old male for the middle-age group (Fig. 4B), and a 64-year-old female for the old group (Fig. 4C). For each subject, on overlay of PAs is shown on cortical surfaces below the corresponding histogram. The overlays for the young subject show greater spatial prevalence of strongly positive PAs (orange areas), which become less prevalent for the middle-aged subject (Fig. 4B), and least for the older subject (Fig. 4C). This age trend is also evident from comparison of their histograms (first row in Fig. 4A-C). Thus, the reduction of pHRF PA variability with age appears to be associated with a moderation of response amplitudes in sensory regions such as lateral occipital (visual) and superior temporal (auditory) areas. This effect is also evident in normalized distributions of PA averaged across the three age groups (Fig. 4D). For pHRFs, the distributions show a small shift of the mode to the right, while a “tail” of strong activation decreases. These two age-dependent effects, which are subtly evident in Fig. 4D, can be quantified by examining the behavior of quintiles of the amplitude distribution (Fig. S3A). The upward trend in the mode is seen in the significant (*p* = 0.006) positive trend in the 20th percentile, while the erosion of the tail with age is evident in the significant (*p* = 0.028) negative trend at the 80th percentile.

For nHRFs, the mean distributions of PAs show a simpler behavior, with the distribution steadily shifting toward greater (more negative) amplitudes with increasing age. Quantifying by quintiles (Fig. S3B), there are significant downward trends in 40th, 60th, and 80th percentiles.

As mentioned above, regression showed a substantial increase in FWHM spatial variability with age (Fig. 3), with a stronger correlation for pHRFs (*R* = 0.52) than nHRFs (*R* = 0.27) (Fig. 3). These results are further understood by examining the FWHM distributions (Fig. 5). FWHM distributions of strong HRFs and their corresponding maps overlaid on gray-white interface surfaces are shown for the same representative subjects used for Fig. 4. Average HRFs (5-mm-diam gray-matter disk) in six example ROIs are plotted for each subject. The young subject (Fig. 5A) had a relatively narrow, unimodal pHRF FWHM distribution with a peak at 4.7 sec. The overlay of FWHM on the cortical surface is consistent with the fairly stable dynamics reported previously^51^. Example HRFs illustrate stereotypical HRF behavior. However, the FWHM distribution for the middle-aged adult was notably broader (Fig. 5B), with many narrower HRFs across cortex evident on the overlay. Example HRFs illustrate the mixture of stereotypically broad as well as narrower HRFs. This broadening of the FWHM distribution toward narrower HRFs is even stronger for the older adult (Fig. 5C), with the distribution becoming strongly bimodal, and the diversity of FWHM values evident on the surface overlay. Example HRFs illustrate these narrow and broad classes of HRFs. FWHM distributions from all subjects were normalized by their peak values and averaged together for young (< 40 years), middle (40–59 years), and old (≥ 60 years) groups (Fig. 5D). Clearly, pHRFs with lower FWHM values are less likely in the young group (blue distribution), becoming more common with advancing age, so that the older group shows a bimodal distribution (red distribution), with a distinct mode of narrow HRFs, as well as a greater representation of unusually broad HRFs. Examination of FWHM distributions for all subjects (Fig. S4) confirm this tendency toward broader, bimodal distributions with increasing age.

Scatter plots of PA versus FWHM show that largest amplitudes are associated with a fairly narrow temporal range for both positive and negative HRFs (Fig. 6A) in the same three example subjects used previously. Tuning curves obtained from this data emphasize this feature and show strong changes with age (Fig. 6B). When these curves are averaged over our three age groups, the age-related changes become clear (Fig. 6C). For positive HRFs, tuning is sharpest for the young group (blue), becoming broader for the middle (green) and older (red) groups. Moreover, the old group exhibits a tendency toward a second peak of faster dynamics. For negative HRFs, the behavior is reversed, with tuning broadest for the young group and becoming sharper for both middle and older groups with a possible second mode of slower dynamics emerging for the older group. The changes in tuning width indeed vary significantly with age (Fig. 6D) for both positive HRFs (*R* = 0.49, *p* < 10^−4^) and somewhat more weakly for negative HRFs (*R* = 0.29, *p* = 0.032).

Finally, we found a significant association between the age and the HFPF of both pHRFs and nHRFs for both spatial mean (Fig. 2) and standard deviation (Fig. 3). In Fig. 7, these age-related trends are illustrated for the same representative subjects used previously. Cortical areas with strong HFPF were lowest for the young subject (note prevalence of dark blue clusters in Fig. 7A), becoming greater for the middle-aged subject (Fig. 7B, increasing prevalence of cyan clusters) and greatest for the older subject (Fig. 7C). Sample HRFs show the character of the high-frequency oscillations in these three subjects.

HRF power spectra were normalized by their peak value at frequencies < 0.1 Hz for all 55 sessions (Fig. 7D). All but one session show spectra that peak at a low frequency with a tail toward higher frequencies. However, for many middle-aged and older subjects, the spectrum also features a smaller peak at a relatively high frequency (> 0.2 Hz). Notably, one session (female, age 58) showed an atypical spectrum with a larger peak at high frequency. When these normalized spectra were averaged over our three age groups (Fig. 7D, middle), the low-frequency spectrum was narrowest for older (red), then middle-aged (green), and broadest for the young (blue). In addition, the power spectra for both middle-age and old groups show a distinct second peak at high frequency.

The trend toward narrower FWHM and greater HFPF are necessarily linked. To investigate, we tested whether the trend between the HFPF and age was different for pHRFs with FWHM in narrow-mode range (1.5–3.5 s) versus those in the wide-mode range (4–6 s) across all subjects (Fig. 7E). For both, correlations between HFPF and age were highly significant. Notably, the age trend was stronger for the narrow-mode FHWM HRFs (slope = 0.075%/year vs. 0.038%/year), but the correlation was stronger for the wide-mode FWHM HRFs (*R* = 0.53, *p* = 0.000022 vs. *R* = 0.43, *p* = 0.001). Moreover, scatter plots of HFPF versus FWHM suggest that large HFPF is associated with narrow FWHM (Fig. 7F). Associated tuning curves confirm a dominant peak near 2.3-s FWHM, with a lesser peak or shoulder near 5-s FWHM (Fig. 7G). This qualitative pattern shows little age dependence, but there is a significant decrease with age in HFPF near the 5-s shoulder (Fig. 7H).

## Discussion

We provide a detailed evaluation of age-related HRF trends using an experimental protocol that activates the majority of cerebral cortex. High-resolution fMRI acquires this data with minimal partial-volume effects in the native space of a group of subjects with ages between 22–75 years. We find significant changes in the distributions of both HRF amplitudes and, more importantly, dynamics. Our main findings are: (1) There is a significant trend for mean CNR to decrease with age, but this trend is substantially reduced when data are smoothed to 8-mm FWHM. (2) The spatial variability of PA and CNR significantly decrease with age. (3) Dynamically, the strongest age-related trend is an increase in FWHM spatial variability with age. (4) HRF amplitudes tend to be narrowly tuned to a particular range of FWHM values, and the tuning width increases significantly with age. (5) There is a significant increase in HF power fraction mean and standard deviation with age, and the HFPF is tuned to narrow FWHM values. The results document how age systematically changes the dynamics of NVC in a fashion consistent with known changes in the cerebral microvasculature.

In the present work, the SAST^51,52^ enabled us to globally examine age-related changes in the HRF. Our study is, therefore, notably different from previous studies that were only focused on specific brain regions (e.g., motor and vision areas)^34,38,43,45,46,53,54^. The suitability of the SAST for a whole-cortex study of hemodynamic responses was indeed confirmed by our results demonstrating strong (CNR > 3) HRFs in 60–90% of cerebral cortex (average 68±8% at full resolution, 87 ± 8% after smoothing to 8-mm FWHM). For those strongly activated areas, the majority of responses were positive HRFs (average 73±8%, Fig. 1D). Interestingly, there was a marginal trend (*p* = 0.15) for an increase in the fraction of activated cortex with strong HRFs after 8-mm FWHM smoothing (Fig. S2A), suggesting that, after smoothing, data reliability increases with age.

The SAST also evokes a substantial fraction of negative HRFs. Moreover, the fraction of negative HRFs shows a marginally significant decrease with age. Because our negative HRFs likely correspond predominantly to brief deactivation of the default-mode network (DMN), this decrease with aging may be consistent with previous work showing disruption of the DMN as a consequence of aging^55,56^. Our results suggest that the disruption is associated with a decrease in deactivated cortical area.

The distributions of positive HRF amplitudes were significantly affected by age in two ways. First, evaluation of the distributions by quintiles (Fig. S3) showed two distinct effects for positive HRFs: weaker amplitudes significantly increase with age, while strong amplitudes significantly decrease with age. Qualitatively, the statistical mode of the pHRF amplitude distributions increase with age, while the tail of strong amplitudes diminishes with age. Second, spatial variability of pHRFs strongly and significantly decreased with age (Fig. 3). The pHRF results suggest that NVC becomes more conservative with aging, with a paucity of high-amplitude HRFs, consistent with previous reports^43,45,46,53,54^. For nHRFs, the behavior is simpler, with all HRFs becoming stronger (more negative) with age, but this effect is clearest for the weakest nHRFs. Taken together with the decrease in nHRF fraction, this suggests more conservative allocation of NVC suppression with aging.

Amplitudes and CNR may have been affected by task performance and head motion. Task performance significantly dropped with age (Figs. 1A,B). Accuracy and validity were probably lower for older adults because they tended to respond too slowly, so that their responses referred to the previous dot display. The decreased performance may have impacted pHRF CNR, which showed a significant downward trend with age (Fig. 2). However, no correlations were found with measured task performance metrics. This decrease in CNR may also have been caused by greater head motion in the older subjects, so that motion censoring decreased the number events available for analysis. However, when we removed this effect by scaling the CNR values up by the square-root of available events, the correlation of CNR with age was not significantly altered. Thus, neither performance nor head motion seems to have played a dominant role in the age-related decreases in PA and CNR. Interestingly, these downward trends weakened with 8-mm FWHM smoothing (Fig. S3), suggesting that the CNR degradation is spatially fine grained. This, however, stands in contrast to the other parameter variations with age, which were unaffected by smoothing (Figs. S3–4). Apparently, smoothing reduces the CNR loss associated with aging, but does not much affect the age trends of the HRF dynamical parameters.

We also examined the effect of aging on HRF dynamics. Our results showed that the spatial variability of FWHM strongly and significantly increases with age (Fig. 3). Moreover, comparison of distributions averaged over young, middle-aged, and older groups (Fig. 5D) show a broadening of the overall distribution as well as the emergence of a distinct mode of narrow-FWHM HRFs. However, a detailed examination of FWHM distributions for all subjects (Fig. S4) indicated that two distinct modes were not always found in older subjects, while a bimodal distribution was also present in a few middle-aged adults. This pattern of results suggests that the associated changes in NVC do not occur in all older subjects. Further experiments are needed to assess the relationship of these dynamical changes to pathologies such as hypertension.

Such findings, to our knowledge, have not previously been reported. In a study by West et al (2019), the averaged HRFs for each occipital, temporal, left hemisphere precentral lobes did not show any significant differences in FWHM between younger and older groups^38^. In another study by Zesse et al. (2007), an increase in HRF FWHM was only found in the inferior frontal junction and the inferior fusiform gyrus for older versus younger groups^57^. It is possible that such changes are not broadly evident when data is evaluated in a standard space or after averaging across large ROIs because the spatial patterns of these narrow FWHM occurrences are not common across subjects. The examples in Fig. 5 support this idea, but this hypothesis needs more extensive testing.

This change in HRF dynamics is consistent with age-dependent microvascular disruptions in the context of linear-systems analysis. Specifically, in the healthy young brain, the pial arterioles provide a pressurized, highly interconnected mesh that enables the NVC that creates the HRF. Disruption of this mesh by arterial rarefaction or lipohyalinosis could cause both faster and slower dynamics. Faster dynamics could correspond to a loss of linkage between pial-mesh elements, thus reducing the available compliance and arterial blood volume to drive NVC. Compliance might also be reduced directly in arteriolar segments by wall thickening and decrease of the luminal diameter. In other words, mesh disruption reduces the stored hemodynamic energy available to respond to a proximal integration event. Such faster and weaker dynamics would correspond to gray-matter regions with a viable penetrating arteriole connection to a disrupted portion of the pial mesh. However, slower dynamics could correspond to gray-matter regions without direct connection to the pial mesh. Such regions might still manifest NVC by blood flow among superficial arteriole anastomoses^58-60^. The longer path lengths for such NVC could create the observed broadening of the HRF FWHM. Such collateral circulation mechanisms have been associated with compensation for focal ischemia from stroke, but they could also provide NVC in the face of age-related disruptions to the pial mesh.

Our results show that HRF dynamics exhibit a tuning that varies significantly with aging. In healthy young subjects, stronger pHRF amplitudes are associated with a fairly narrow range of FWHM values that peaks near 5 s. These observations are consistent with our earlier results that showed stereotypical HRF dynamics across cortex^51^. For pHRFs, the tuning broadens with age, again possibly reflecting disruption of the pial arteriole mesh. The disrupted mesh exhibits additional dynamics, both faster and slower, that deliver weaker HRFs and NVC. However, negative HRFs become more tightly tuned with age. Because the majority of our negative HRFs correspond to deactivations of the DMN, this may again be related to age-related decreases in DMN activation^61,62^.

We also report, for the first time, stimulus-evoked high-frequency oscillations in the HRF. The observed dynamics are inconsistent with noise. Our analyses were limited to voxels with strong (CNR > 3) HRFs. Moreover, the power spectra for many older and middle-aged adults demonstrated a distinct peak in range of 0.2–0.4 Hz, consistent with a coherent oscillatory feature. The consistent oscillations that are evident after averaging over ~ 80 events could not be manifest by noise or physiological nuisance. Thus, the advent of greater HFPF with age appears to indicate a new dynamic that can emerge with aging. These results provide a hemodynamic basis for the findings of previous studies exploring age-related changes in the frequency of resting-state fMRI signals^63,64^. In particular, Zhong (2022) demonstrated spatially extensive increases in the 0.1–0.3-Hz range.

The HF features could reflect fundamental modes of the pial mesh. From a linear-network viewpoint, when the mesh is intact and roughly balanced, many of the poles and zeroes of the network cancel out, leaving the observed low-frequency dynamics that are tuned to a FWHM near 5 s. When the mesh is abrogated by aging or age-related pathology, faster oscillations could emerge. The tuning of the oscillations toward the narrow-FWHM dynamics with age may be consistent with a weakly damped flow response suggested by our previous modeling^3,10^. Further experiments and simulations will be needed to test this hypothesis.

Our study has several limitations. First, we did not deal with sex differences among our subjects. In fact, our subject pool was not well sex balanced, with more females than males. Future work will be necessary to evaluate this issue. Second, we utilized 55 sessions from 34 subjects, which reduces the independence of some of the data in a fashion that is difficult to control. Further experiments that expand this dataset to confirm the observed significant trends would be useful. Finally, these data relied on BOLD contrast to evaluate age effects. Further experiments with time-resolved HRF flow measurements^65^ would be desirable.

Normal aging has been associated with increases in flow pulsatility due to mismatch between peripheral arterial stiffness and aorta stiffness.^66-69^ Such changes are exacerbated by hypertension, which occurs increasingly with age. The pulsatility can damage the cerebral microvasculature. Therefore, we hypothesize that excessive flow pulsatility in older adults contributes to disruption of the pial mesh, consistent with weaker, more oscillatory HRFs with both narrower and broader FWHM. Future work is needed to assess whether these dramatic age-related changes in HRF dynamics and NVC are associated with hypertension or other age-related pathologies. Nevertheless, use of the SAST already shows great promise as a non-invasive metric for age-related pathology.

## Methods

### Participants

A cohort of 37 healthy subjects (ages: 22–75 years) with no history of neurological diseases was recruited for this study. Of those, 23 subjects performed two scanning sessions in the same day separated by three hours; this data was part of a study of HRF temporal stability published previously.^52^ For the other subjects, only one session was collected, thereby yielding a total number of 60 datasets. Five sessions were then excluded from analysis due to excessive motion or low contrast-to-noise ratio. From the remaining 34 subjects (20 female), we analyzed 55 scanning sessions including 23 young (22–39 years, mean: 26.9 years, 8 male), 14 middle-age (40–59 years, mean: 52.4 years, 12 male), and 18 older (60–75 years, mean: 65.9 years, 5 male) adults. These age groups were chosen as a convenient 3-way split that roughly balances the available pool of subjects and sessions. For each subject, informed consent form was obtained at the time of first enrollment in the study. All experimental procedures were performed according to a protocol reviewed and approved by the Baylor College of Medicine Institutional Review Board. All the methods described here were in accordance with relevant institutional guidelines and regulations. Prior to data collection, each subject was trained on the task and screened for MRI safety.

### Experimental Tasks

**Figure 8:**
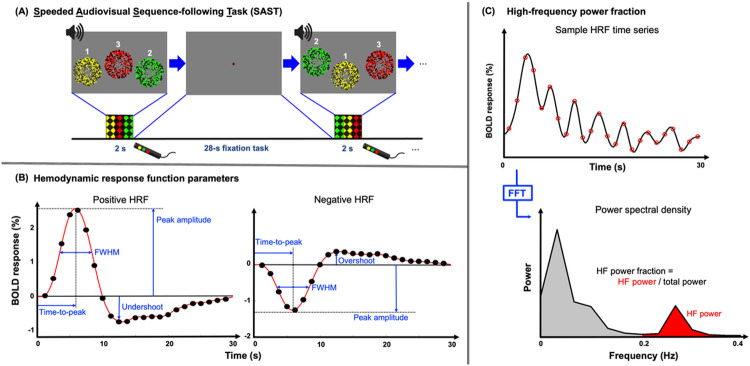
(**A)** Schematic shows the SAST sequence. A single HRF event consists of the 2-second stimulus task and the 28-second resting period fixation task. (**B)** HRF parameters are extracted from positive and negative BOLD responses including HRF peak amplitude, peak time, FWHM, and under/over-shoot amplitude. (**C**) High-frequency power fraction is calculated as the ratio of high-frequency (>0.2 Hz) power to total power.

HRFs are evoked by a 2-second speeded audiovisual sequence-following task (SAST) followed by a 28-second non-demanding fixation task (Fig. 8A). Stimulus onset is cued by a change of fixation dot color for 0.5 seconds before each 2-second stimulation period. The stimulus has three components: visual, audio, and task. Visual stimulation consists of three consecutive presentations of flickering (6 Hz) colored dots, half brightly colored and half darkly colored to enhance contrast. The dots are presented in one of three circular (5° radius) regions for 667 ms. The regions are uniformly distributed horizontally across the width of the display with each position having a specific color: yellow on the left, green in the center, and red on the right. Vertical positions are randomly varied over a ± 3° range. The spatial order of presentation is random without sequential repetition. Each dot-region display is accompanied by an audio stimulus of filtered white noise: medium pitch during yellow dots; low pitch for red; high pitch for green. Subjects are instructed to follow the colored dot regions with eye movements and quickly (within 667-ms) press a button that matched the position, color, and sound presented. Reaction time and response accuracy are tracked during each run. Between these strong audiovisual stimuli, subjects perform a non-demanding fixation task to maintain fixation and a low level of attention. During the fixation period, subjects attend the central-colored dot (0.15° diameter) that changes color every 0.6 s. Subjects press a button at the appearance of a single target color, which appears on average every 6 seconds^51^. A stimulus and a fixation period constitute a 30-second HRF measurement. Sixteen HRFs are collected in each of 5 runs to yield 80 HRF measurements for each session.

### Magnetic Resonance Imaging Protocol

Imaging was performed on a 3T MAGNETOM Trio MRI scanner (Siemens Healthcare, Erlangen, Germany) using a 32-channel RF head coil. FMRI data use an SMS-accelerated echo-planar imaging (EPI) sequence^70,71^ with TR = 1.25 seconds, TE = 30 ms, GRAPPA = 2, SMS = 3, 2-mm square pixels on 60 2-mm slices. During functional runs, stimulus timing aligns with the TR of the scanner (24 TRs per HRF).

During each session, a T1-weighted volume aligned with the functional slice prescription was acquired before and after collecting functional images using a 3D FLASH sequence with minimum TE and TR, 15° flip angle, 256-mm FOV, 63 slices, 2-mm slice thickness, and 1-mm inplane pixel size. There images were used to align the functional data to a high-resolution T1 -weighted reference volume. The reference volume was obtained for each subject using a high-resolution (0.8-mm isotropic voxels) MP-RAGE sequence with TR = 2300 ms, T1 = 900 ms, flip angle = 9°, 2 repetitions. The anatomy was then segmented into gray and white matter using FreeSurfer^72^ with procedures to maintain native spatial resolution.^73,74^ A depth coordinate normalized to local gray-matter thickness was also computed from the segmentation.^50^

### Data Analysis

For each fMRI scan, images were compensated for slice acquisition timing and for the effects of head motion using a robust expectation-maximization method.^75^ Next, the motion-corrected functional data were then brought into spatial alignment with each other and finally registered to a high-resolution reference volume anatomy using the same intensity-based alignment algorithm applied to the T1-weighted inplane images collected in each session. Low-frequency temporal baseline drifts were also removed from the time series, and the data were corrected for spatial variations due to receiver-coil-array inhomogeneity.^76^

For each subject’s reference volume, we created a normalized distance map (0 on the white-matter surface to 1 on the pial surface), which also provides streamlines that uniquely associate all GM voxels to the white-matter surface.^50^ To minimize partial volume effects, functional data of voxels located in the normalized depth range of 0.2–0.8 were then averaged together and mapped onto the white-matter surface vertices.^50^

The surface data was also smoothed with an 8-mm-FWHM Gaussian kernel using surface-manifold coordinates. Larger clusters of nHRFs (> 36 mm^2^) were smoothed separately to avoid mixing between nHRFs and pHRFs. Both full-resolution and smoothed HRF data were parameterized and analyzed for age-related effects.

For each subject, the depth-averaged data were censored for excessive motion, so that any of 80 (16 events per scan x 5 scans) HRF events with any head motion > 2 mm/TR was removed from further analysis. The HRF at each surface vertex was finally created by averaging the surviving event and was characterized by four parameters (Fig. 8B): (1) peak amplitude (PA), (2) time-to-peak (TTP), (3) full-width-half-maximum (FWHM), and (4) undershoot amplitude. PA was forced to be in the interval 2–14 s to reduce outliers from occasional noise in the time series. Before parameterization, a zero baseline was estimated for each HRF as the average of its first and last time points, which was then subtracted from the time series. Each HRF’s time series was then unsampled to an interval of 0.1 s. In addition to the four parameters, peak contrast-to-noise ratio (CNR) and high frequency power fraction (HFPF) were obtained for each HRF. The HRF’s CNR was calculated as the ratio of its PA (before upsampling) to its standard error of the mean (SEM) across the events. HFPF was defined as the ratio of the power in the upper half of the frequency range, 0.2–0.4 Hz (Nyquist frequency is 0.4 Hz) to the total power (Fig. 8C). Finally, to characterize the global tuning of HRFs across cortex for each subject, we evaluated the dependence of PA or HFPF on FWHM. These tuning curves were obtained by summing PAs or HFPF within small ranges of FWHM across the domain of 1–11 s, thus providing two further measures of hemodynamic energy as a function of HRF time-scale. These tuning curves were then normalized by their peak value to permit comparisons among subjects. The FWHM width of the tuning curves, a tuning width, was used as a single parameter for evaluation as a function of age.

Based on the sign of the PAs, the HRFs were divided into positive (pHRFs) and negative HRFs (nHRFs). For both, the spatial mean and standard deviation of all parameters, as well as CNR and HFPF were computed. We chose only strong HRFs with CNR > 3 for analysis. We then performed regression analysis to assess statistically significant (*p* < 0.05) correlations between HRF parameters and age.

## Data Availability

Processed data, HRF time series and their parameters, mapped to native-space vertices in NIFTI format, will be made available upon request to the corresponding author.
